# Fimbrin associated with Pmk1 to regulate the actin assembly during *Magnaporthe oryzae* hyphal growth and infection

**DOI:** 10.1007/s44154-023-00147-5

**Published:** 2024-01-22

**Authors:** Yuan-Bao Li, Ningning Shen, Xianya Deng, Zixuan Liu, Shuai Zhu, Chengyu Liu, Dingzhong Tang, Li-Bo Han

**Affiliations:** 1 State Key Laboratory of Ecological Control of Fujian-Taiwan Crop Pests, Key Laboratory of Ministry of Education for Genetics, Breeding and Multiple Utilization of Crops, Plant Immunity Center, Fujian Agriculture and Forestry University, Fuzhou, Fujian China; 2 College of Agriculture, Fujian Agriculture and Forestry University, Fuzhou, Fujian China; 3 College of Life Sciences, Fujian Agriculture and Forestry University, Fuzhou, Fujian China; 4 School of Future Technology, Fujian Agriculture and Forestry University, Fuzhou, Fujian China

**Keywords:** *Magnaporthe oryzae*, Fimbrin, Pmk1, Actin cytoskeleton

## Abstract

The dynamic assembly of the actin cytoskeleton is vital for *Magnaporthe oryzae* development and host infection. The actin-related protein MoFim1 is a key factor for organizing the *M. oryza*e actin cytoskeleton. Currently, how MoFim1 is regulated in *M. oryzae* to precisely rearrange the actin cytoskeleton is unclear. In this study, we found that MoFim1 associates with the *M. oryzae* mitogen-activated protein (MAP) kinase Pmk1 to regulate actin assembly. MoFim1 directly interacted with Pmk1, and the phosphorylation level of MoFim1 was decreased in Δ*pmk1*, which led to a change in the subcellular distribution of MoFim1 in the hyphae of Δ*pmk1.* Moreover, the actin cytoskeleton was aberrantly organized at the hyphal tip in the Δ*pmk1*, which was similar to what was observed in the Δ*mofim1* during hyphal growth*.* Furthermore, phosphorylation analysis revealed that Pmk1 could phosphorylate MoFim1 at serine 94. Loss of phosphorylation of MoFim1 at serine 94 decreased actin bundling activity. Additionally, the expression of the site mutant of MoFim1 S94D (in which serine 94 was replaced with aspartate to mimic phosphorylation) in Δ*pmk1* could reverse the defects in actin organization and hyphal growth in Δ*pmk1*. It also partially rescues the formation of appressorium failure in Δ*pmk1.* Taken together, these findings suggest a regulatory mechanism in which Pmk1 phosphorylates MoFim1 to regulate the assembly of the actin cytoskeleton during hyphal development and pathogenesis.

**Supplementary Information:**

The online version contains supplementary material available at 10.1007/s44154-023-00147-5.

## Introduction

Rice blast caused by the fungus *Magnaporthe oryzae* is one of the most destructive diseases of rice and is a main crop nourishing more than half of the world’s population. A great amount of harvest loss occurs in all rice-growing regions of the world every year (Wilson and Talbot 2009). The basic unit of growth of *M. oryzae* is the hypha, which consists of tube-like structures with a hemispherical or hemiellipsoidal apical region (Fernandez and Orth 2018). The polar growth of *M. oryzae* hyphae is a fundamental property for cell development and is accompanied by the transport of cellular components to specific sites in the cell. It requires the continuous synthesis and selective targeting of proteins, lipids, and cell wall materials to specific domains at the apex of the hyphal plasma membrane (Riquelme 2013). During *M. oryzae* infection, the polar growth of hypha occurring at the hyphal tip is associated with endocytosis and effector secretion, which is critical for the invasive hypha extending across the plasmodesmata to spread in the host plant cell (Wendland and Walther 2006). Thus, understanding the biology of fungal cells is required for revealing the infection mechanism of *M. oryzae.*

Mitogen-activated protein (MAP) kinase cascades play important roles in environmental signal sensing, development and host infection in plant fungal pathogens. The MAPK signaling pathway is important for plant infection in more than 20 plant pathogenic fungi (Li et al. 2012). It has been revealed that in *M. oryzae*, a mitogen-activated protein kinase (MST11-MST7-Pmk1) MAP kinase cascade exists that guides infection-related morphogenesis (Zhao et al. 2005). A MAPK docking site in Mst7 is essential for Pmk1 activation during *M. oryzae* infection (Zhao and Xu 2007). Mst50 functions as an adapter protein of the Mst11-Mst7-Pmk1 cascade that is essential for appressorium formation (Park et al. 2006; Li et al. 2017a). In the model organism *Saccharomyces cerevisiae,* there are five MAPK proteins (Fus3, Kss1, Slt2, Hog1, and Smk1) (Waltermann and Klipp 2010; Xu and Hamer 1996). However, in most plant pathogenic fungi, only three MAPK genes orthologous to Hog1, Fus3/Kss1, and Slt2 (Jiang et al. 2018) are present. Pmk1 is an orthologue of Fus/Kss1 and was suggested to be critical for fungal development and appressorium formation in appressorium-forming fungi, including the rice blast fungus *M. oryzae*, *Colletotrichum lagenarium*, and *Colletotrichum gloeosporioides* (Xu and Hamer 1996; Takano et al. 2000; He et al. 2017; Abah et al. 2023). Mutants of *PMK1* in *M. oryzae* fail to construct specialized infection structures known as appressorium and fail to develop invasive hyphae in rice plants (Xu and Hamer 1996). Detection of green fluorescence protein (GFP)-labelled Pmk1 showed that Pmk1 was expressed in vegetative hyphae, conidia, and germ tubes and that the expression of GFP-Pmk1 increased in appressorium and developing conidia. Especially in the appressorium, GFP-Pmk1 fluorescence can be observed in the nucleus (Bruno et al. 2004), indicating the roles of Pmk1 in these processes. The *M. oryzae PMK1* mutant could not develop a mature appressorium from conidial germlings on a hydrophobic surface (Xu and Hamer 1996). Lipid and glycogen mobilization and autophagy were disrupted in the *PMK1* mutant (Thines et al. 2000; Veneault-Fourrey et al. 2006). The *PMK1* mutant hyphae could not penetrate the neighboring host cell and thus failed to undergo cell-to-cell movement (Sakulkoo et al. 2018). Recently, Pmk1 was shown to trigger the infection process through phosphorylation of the Hox7 homeobox transcription factor, which is associated with the development of *M. oryzae* appressorium, including cell division, autophagy, turgor generation and melanin biosynthesis (Oses-Ruiz et al. 2021). These findings indicated that Pmk1 is particularly important for *M. oryzae* infection, and the complex molecular mechanism underlying Pmk1 infection has become a hot topic in *M. oryzae* research.

The actin cytoskeleton plays a central role in cell morphogenesis and maintaining hyphal polar growth in filamentous fungi (Berepiki et al. 2011). Three high-order filamentous actin (F-actin) structures with distinct functions, actin rings, patches, and cables, were found to be organized in *M. oryzae*. The actin rings are suggested to associate with the formation of a septum, and the actin patches localize to subapical regions and are involved in endocytosis. Actin cables are linear bundles of short actin filaments that are present at the apexes of hyphae (Riquelme 2013). At the hyphal tip of *M. oryzae*, the actin cytoskeleton is dynamically assembled as a spatial structure that guides the growth of the cell (Li et al. 2020). The assembly of actin is guided by numerous actin-related proteins, such as motor proteins, actin bundle proteins, and septins. Mutants of these actin-related protein genes in *M. oryzae* exhibit severe defects in either development or host infection (Guo et al. 2017; Motaung and Tsilo 2017; Tang et al. 2018; Xu et al. 2021). Fimbrin is a typical actin bundle protein, and *M. oryzae* fimbrin are known to localize to the hyphal tip region (Gupta et al. 2015) and to regulate actin assembly at the hyphal tip during polar growth. Knockout of fimbrin (MoFim1) in *M. oryzae* could abolish the infection ability (Li et al. 2020). Although we know the importance of MoFim1 in *M. oryzae* development and pathogenesis, we do not know how it is regulated.

In this study, we found that *M. oryzae* MoFim1 could directly interact with Pmk1 and that Pmk1 could affect the phosphorylation level of MoFim1. Phosphorylation of MoFim1 was decreased in the Δ*pmk1* strain. Dephosphorylation of MoFim1 resulted in altered localization and decreased actin bundling activity, thus affecting the organization of the actin cytoskeleton in hyphae. We provide evidence that Pmk1 could regulate the actin cytoskeleton through modulation of the phosphorylation of the actin-related protein MoFim1 during *M. oryzae* development and pathogenesis.

## Results

### MoFim1 interacts with Pmk1

**Fig. 1:**
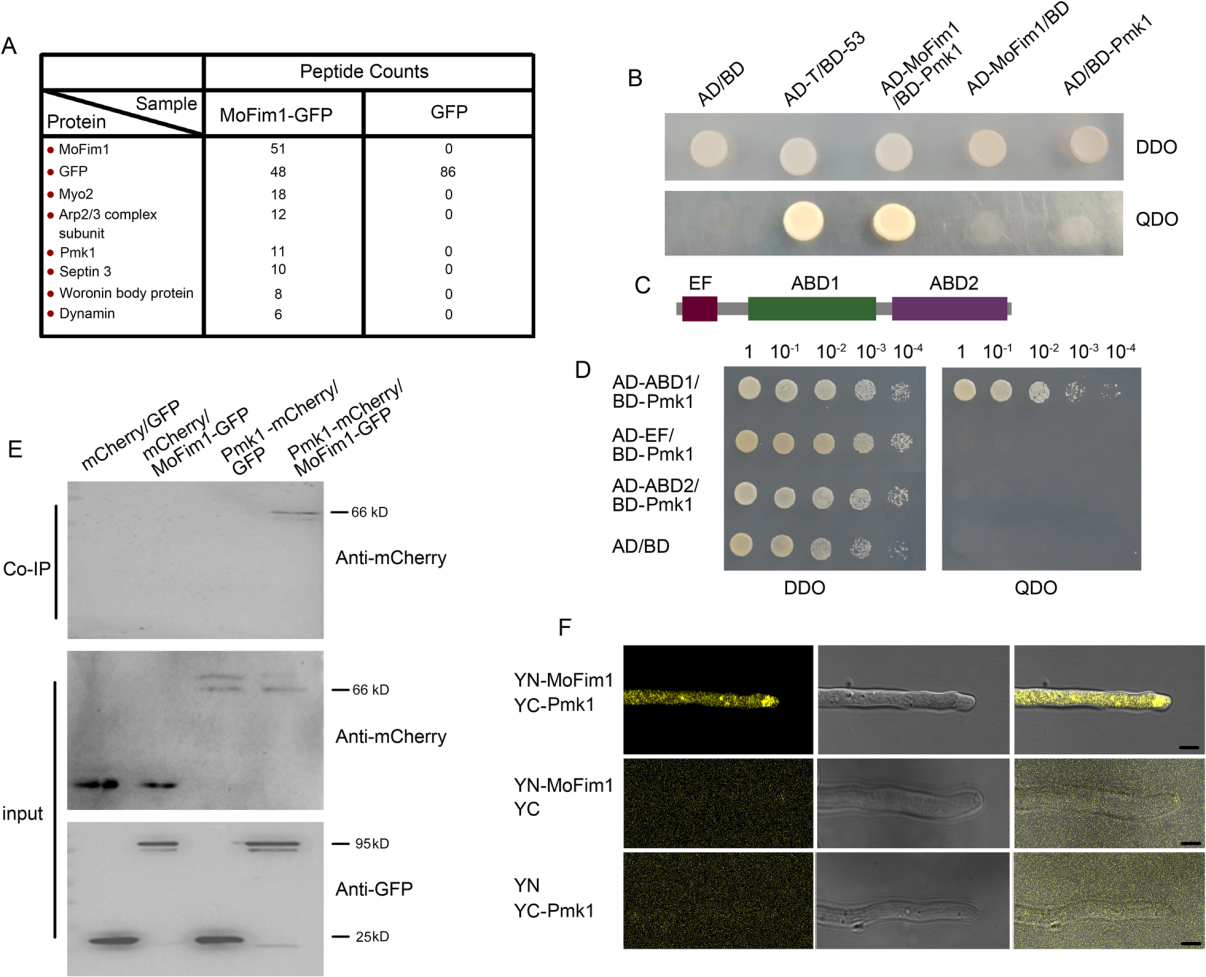
MoFim1 interacts with Pmk1. **A** *M. oryzae* hyphae expressing MoFim1-GFP or GFP were subjected to immunoprecipitation and mass spectrometry (IP-MS). Hyphae of MoFim1-GFP and GFP were collected three times and combined for protein extraction and IP-MS analysis. The most accumulated putative interacting proteins in *M. oryzae* identified by IP with MoFim1 are listed. **B** Yeast two-hybrid assay showing the interaction between MoFim1 and Pmk1. Yeast cells containing the indicated plasmids were grown on SD/-Leu/-Trp DO (DDO) plates and SD/-Leu/-Trp/-Ade/-His DO (QDO) plates. AD-T and BD-53 were used as the negative and positive controls, respectively. **C** Schematic diagram showing the protein domains of MoFim1. **D** Yeast two-hybrid assay showing the interaction sites between MoFim1 and Pmk1. The indicated domains of MoFim1 were expressed in yeast cells and subsequently grown on SD/ − Leu/ − Trp DO (DDO) plates and SD/ − Leu/ − Trp/ − Ade/ − His DO (QDO) plates. **E** Co-IP experiment showing the interaction between MoFim1 and Pmk1. *M. oryzae* expressing the indicated plasmids were used in these experiments. *M. oryzae* proteins were incubated with anti-GFP agarose beads. Immunoblotting was subsequently conducted with anti-mCherry antibodies. **F** Bimolecular fluorescence complementation (BiFC) assays showing the interactions between MoFim1 and Pmk1. YFP, (N)-Fim1 and YC-Pmk1 plasmids were constructed and expressed in wild-type *M. oryzae*. Coexpression of YN and CN was used as a negative control. Bars = 5 μm

Previously, we found that the *M. oryzae* fimbrin protein MoFim1 is an actin-binding protein that is required for the endocytosis, growth, development, and full virulence of *M. oryzae* (Li et al. 2020). To further explore the mechanism by which MoFim1 functions, we employed protein immunoprecipitation mass spectrometry (IP-MS) to identify putative MoFim1-interacting proteins. By expressing the MoFim1-GFP and GFP constructs and using GFP beads to isolate MoFim1-GFP-interacting proteins followed by mass spectrometry (MS) analysis, we identified six proteins that may interact with MoFim1 including the actin motor protein Myosin 2, the Arp2/3 complex subunit protein, Septin 3, Pmk1, the Woronin body protein (Hex1) and a dynamin protein (Fig. 1A). As Pmk1 is vital to *M. oryzae* (Xu and Hamer 1996; Sakulkoo et al. 2018), we next verified the interaction between Pmk1 and MoFim1. We employed the yeast two-hybrid assay (Y2H) to evaluate the interactions. Yeast cells expressing the AD-MoFim1 and BD-PMK1 constructs were grown on SD/-Leu/-Trp DO (DDO) plates and SD/-Leu/-Trp/-Ade/-His DO (QDO) plates (Fig. 1B). The results showed that Pmk1 and MoFim1 could interact in this system. MoFim1 contains three motifs, namely, the N-terminal EF-hand (EF) motif and two actin-binding domains (ABD1 and ABD2), in tandem (Fig. 1C). We further found that Pmk1 could interact with the ABD1 motif (Fig. 1D). We next used a co-IP experiment to verify the interaction of the two proteins. We then expressed MoFim1-GFP and Pmk1-mCherry driven by their native promoters in *M. oryzae* via co-IP (Supplemental Fig. 1). The results showed that Pmk1 coimmunoprecipitated with MoFim1 (Fig. 1E). In addition, bimolecular fluorescence complementation (BiFC) assays further substantiated the interaction between MoFim1 and Pmk1 (Fig. 1F). We noticed that the interactions took place at the hyphal tip area. We also investigated the subcellular distribution of Pmk1. The results showed that Pmk1 was expressed in *M. oryzae* hyphae, the germ tube and the appressorium (Supplemental Fig. 2). Taken together, these results indicate that MoFim1 can interact with Pmk1.

**Fig. 2:**
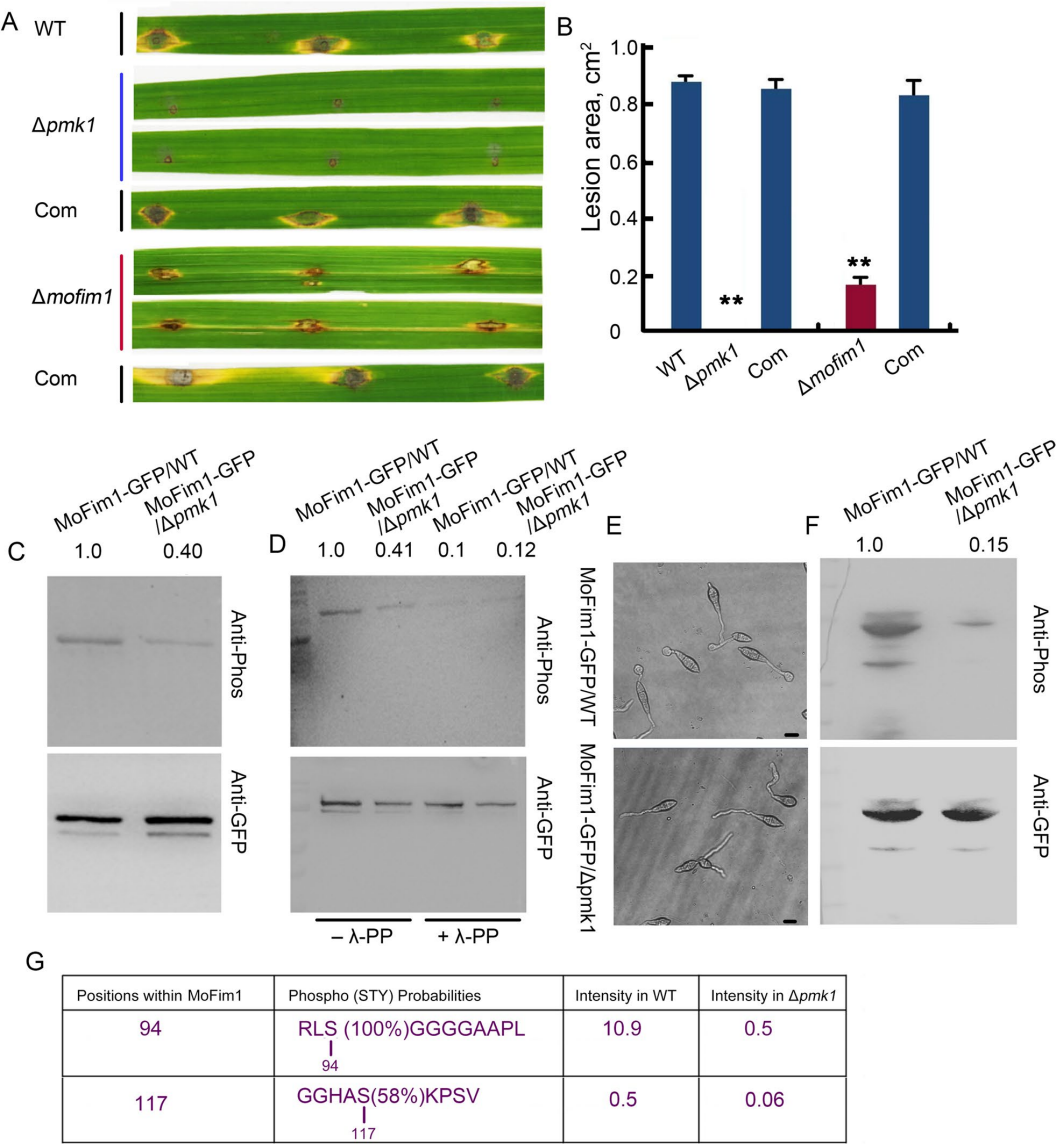
Pmk1 functions in the phosphorylation of MoFim1. **A** Pathogenicity assay of the generated Δ*pmk1*, Δ*Mofim1* and complemented strains after punch inoculation. The same area of each SRB culture plate from the Δ*pmk1*, Δ*Mofim1* and complemented strains was used to infect these rice leaves (*O. sativa cv.* Nipponbare). Photographs were taken 5 days after infection. **B** Quantification of the lesion area on the rice leaves shown in (**A**). The error bars represent SD (*n* = 20), and the asterisks (**) represent significant differences (*P* < 0.01). **C** and **F** Phosphorylation analysis of MoFim1 in Δ*pmk1* hyphae (**C**) and appressorium (**F**)*.* Total proteins from Δ*pmk1* and WT hyphae or 8 h appressorium expressing MoFim1-GFP were extracted and incubated with anti-GFP agarose beads. After washing, the samples were analysed with anti-GFP and anti-phosphorylation antibodies. The number in the figure indicates the fold change in intensity of the band analyzed by ImageJ software. **D** λ-PP was added to test the phosphorylation of MoFim1. The extracted proteins were incubated with λ-PP before western blotting. The number in the figure indicates the fold change in intensity of the band analyzed by ImageJ software. **E** Images of appressourim of WT and Δ*pmk1* used for protein extraction. Bars = 10 µm. **G** Phosphorylation analysis of MoFim1 in the WT and the Δ*mofim1*

### Phosphorylation of MoFim1 was decreased in the Δ*pmk1*

A mutant of *MoFim1* was previously generated in our laboratory (Li et al. 2020). We then generated mutant of *PMK1* in *M. oryzae* to further study their relationships. The generation of a *PMK1* knockout mutant in *M. oryzae* was performed (Supplemental Fig. 3). Next, we tested the infection ability of Δ*pmk1* and Δ*mofim1*, which we previously obtained (Li et al. 2020). We infected the rice leaves in the same region on the plate for the SRB-grown cultures of the WT, Δ*pmk1*, and Δ*mofim1* and their complemented strains. The results showed that the deletion of *PMK1* completely abolished the infection ability, as previously reported (Xu and Hamer 1996; Sakulkoo et al. 2018), and that the Δ*mofim1* mostly lost its ability to infect. The complemented strains could rescue their infections (Fig. 2A and B).

**Fig. 3:**
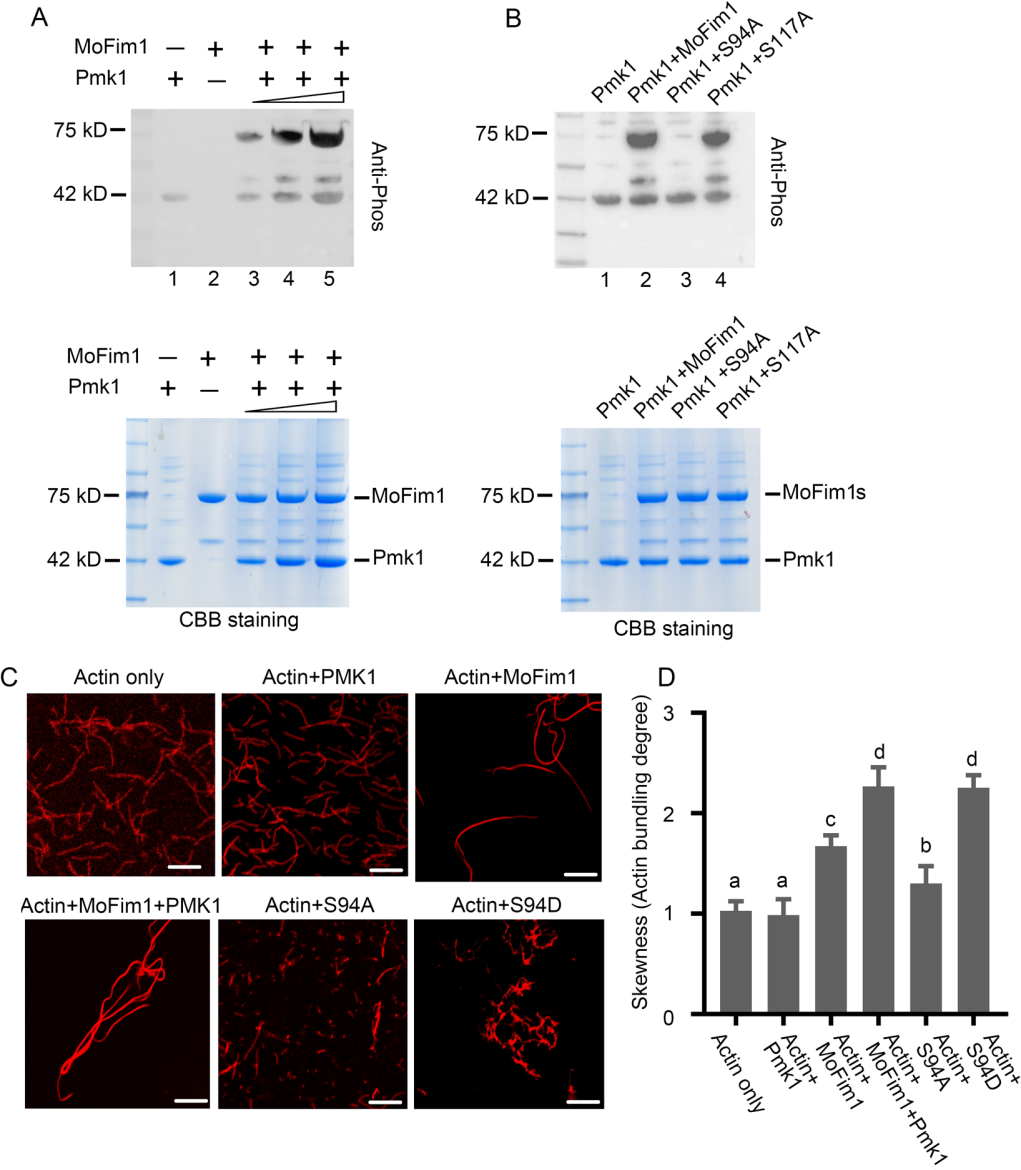
Pmk1-mediated phosphorylation of MoFim1 and actin bundling activity of MoFim1 mutants. **A** Pmk1 phosphorylates Pmk1 in vitro. Recombinant His-Pmk1 was incubated with His-MoFim1 in kinase buffer and detected by immunoblotting with an anti-pSpT antibody that could specifically detect phosphorylated Ser and Thr. Lane 1: 3 µM Pmk1 only; lane 2: 3 µM MoFim1 only; lanes 3–5: increasing amounts of Pmk1 3, 4 and 5 µM Pmk1 incubated with 3 µM MoFim1. **B** Lane 1: 3 µM Pmk1 only; lane 2: 3 µM MoFim1 only; lane 3: 3 µM S94A incubated with 3 µM PmK1; lane 4: 3 µM S117A incubated with 3 µM Pmk1. **C** Actin bundle activity of MoFim1. F-actin was stained with TRITC-phalloidin and observed via fluorescence microbiology. F-actin (3 µM) alone or in combination with the indicated proteins (2 µM). Bars = 2 µm. **D** Statistical analysis of the extent of actin bundles in (**C**)

We next expressed MoFim1-GFP driven by its native promoter in the WT and Δ*pmk1* strains*.* Total proteins from hyphae were examined and incubated with anti-GFP beads. Then, Western blot analysis was performed using both a GFP antibody and a phosphorylation antibody. The results indicated that MoFim1 could be phosphorylated. In addition, phosphorylation of MoFim1 was decreased in the Δ*pmk1* compared with the WT (Fig. 2C)*.* We next added lambda protein phosphatase (λ-PP) to test its phosphorylation. The results showed that phosphorylation of MoFim1 decreased markedly, indicating confidence in the phosphorylation (Fig. 2D). We also analyzed the phosphorylation of MoFim1 in the appressorium. Δ*pmk1* could not form an appressorium (Fig. 2E). Moreover, phosphorylation of MoFim1 in the *pmk1* appressorium significantly decreased compared with that in the WT (Fig. 2F). We further analyzed the phosphorylation of the proteins in both the WT and the Δ*mofim1* by mass spectrometry*.* The results showed that the phosphorylation of MoFim1 at Ser94 and Ser117 was markedly lower in the Δ*pmk1* strain than in the WT strain (Fig. 2G).

### Pmk1 phosphorylated MoFim1 at Ser94, which is critical for its actin bundling activity

To verify that Pmk1 phosphorylates MoFim1, we expressed and purified His-tagged Pmk1 and MoFim1 proteins, which included S94A (with the MoFim1 94 serine changed to alanine to mimic dephosphorylation), S94D (with the MoFim1 94 serine changed to aspartic acid to mimic phosphorylation) and S117A (with the MoFim1 117 serine changed to alanine to mimic dephosphorylation), in *E. coli* BL21 (DE3) cells. Pmk1 alone or incubated with MoFim1 was analysed using a phosphorylation antibody. The results showed that Pmk1 could autophosphorylate (Fig. 3A, lane 1). In the presence of Pmk1, MoFim1 was phosphorylated. Along with the increasing amount of Pmk1, the phosphorylation of MoFim1 increased (Fig. 3A, lanes 3–5). Next, we incubated Pmk1 with S94A and S117A, and the results showed that S94A but not S117A lost phosphorylation (Fig. 3B, lanes 3 and 4). These results indicated that Pmk1 could regulate the phosphorylation of MoFim1 at serine 94.

As the main function of MoFim1 is to bundle the actin cytoskeleton, we next investigated whether phosphorylation of MoFim1 affects its actin bundling activity. We then incubated filamentous actin (F-actin) with these purified MoFim1 or Pmk1 proteins. The results showed that F-actin exhibited thin filaments (Fig. 3C), and incubation with Pmk1 did not affect its polymerization. When MoFim1 was added to F-actin, actin bundles appeared. Furthermore, the presence of Pmk1 or S94D induced thicker actin bundles than did the presence of MoFim1 alone. However, S94A exhibited weaker actin bundle activity than MoFim1. The statistical analysis further supported these conclusions (Fig. 3D). Taken together, these results showed that phosphorylation of MoFim1 at serine 94 is critical for its actin bundling activity.

### Localization of MoFim1 was changed in the Δ*pmk1*

**Fig. 4:**
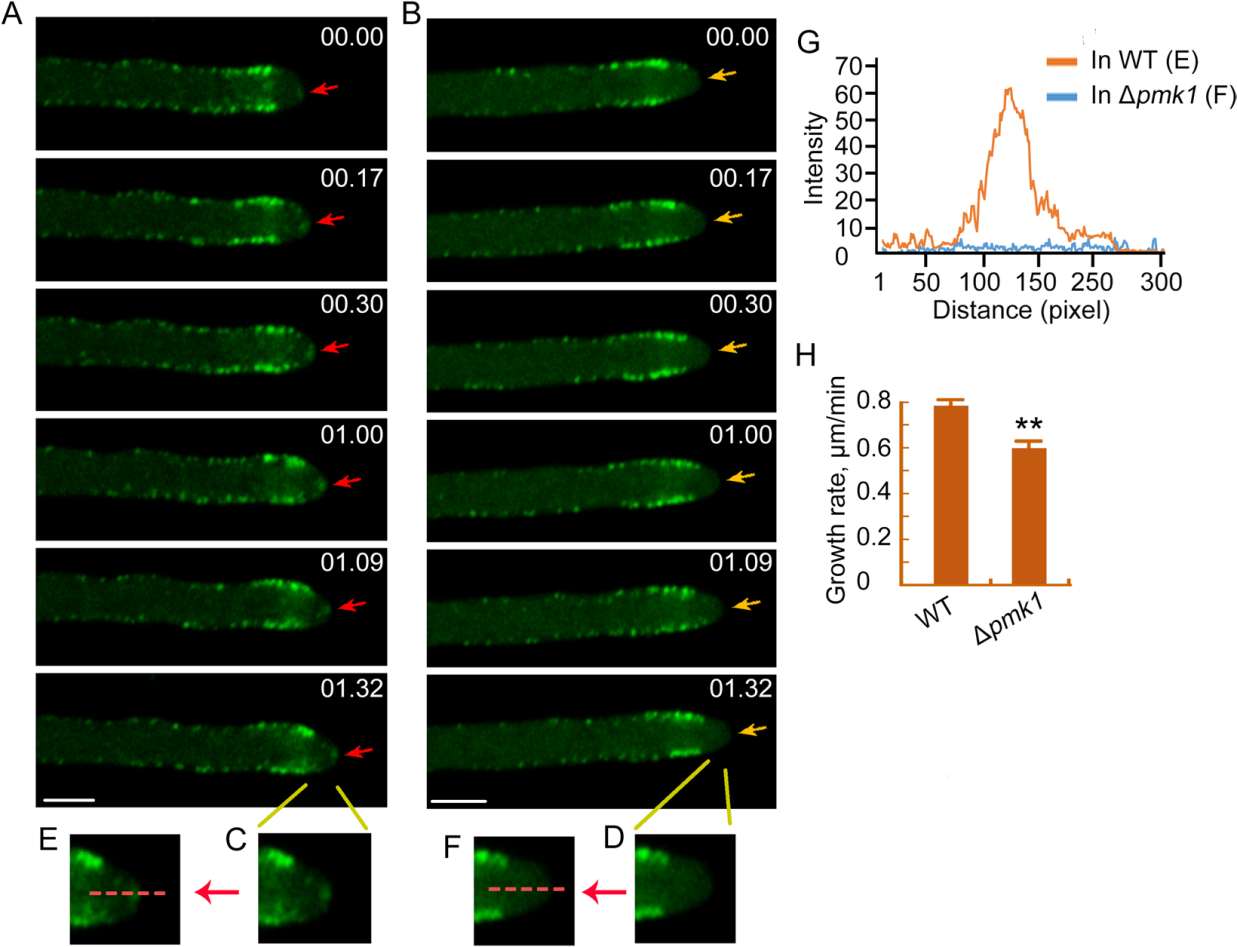
Localization and actin bundling analysis of MoFim1. **A** and **B** Expression of *pMoFim1-MoFim1-GFP* in the WT (**A**) and Δ*pmk1* (**B**) strains*.* The obtained hyphae were observed using high-resolution imaging. The numbers at the top right corner indicate the timestamps (min:s). Bars = 2 μm. **C** and **D** Enlarged region at the hyphal tip. **E** and **F** Line scan analysis of the discrepancy in the localization of MoFim1-GFP in the hyphal tips of the WT and Δ*pmk1.* **G** Line scan analysis of the fluorescence in (**E**) and (**F**). **H** Growth rate analysis of the WT and Δ*pmk1* hyphae according to the observed living hyphae. The data are presented as the mean ± SE; *n* ≥ 30 cells. Asterisks indicate statistically significant differences according to Student’s *t* test (**p < 0.01)

The above results showed that the phosphorylation of MoFim1 was compromised when *PMK1* was knocked out. We then checked the cellular localization of MoFim1 in both the WT and Δ*pmk1.* Time-lapse imaging of vegetative hyphae revealed that MoFim1-GFP formed dense patches in the cytoplasm, the collar in the subapical region and the Spitzenkörper (Spk) region at the hyphal tip. (Fig. 4A, Supplemental Movie 1). We then investigated distribution of MoFim1 in Δ*pmk1.* We found that, in the hyphal subapical region, the localization of MoFim1 did not change significantly. However, in the hyphal tip, the distribution of MoFim1 was abolished (Fig. 4B, Supplemental Movie 2). In the enlarged regions of the hyphal tip, the distribution of MoFim1-GFP in the Δ*pmk1* was lost compared with that in the WT (Fig. 4C and D). The line scan analysis further supported these findings (Fig. 4E, F and G). We also found that the hyphal growth rate decreased in the Δ*pmk1* (Fig. 4H)*.* Taken together, these results indicated that Pmk1 is required for the hyphal tip localization of MoFim1.

### Actin organization was disrupted in Δ*pmk1, *and Δ*mofim1 *and S94D could reverse the actin defects in Δ*pmk1*

**Fig. 5:**
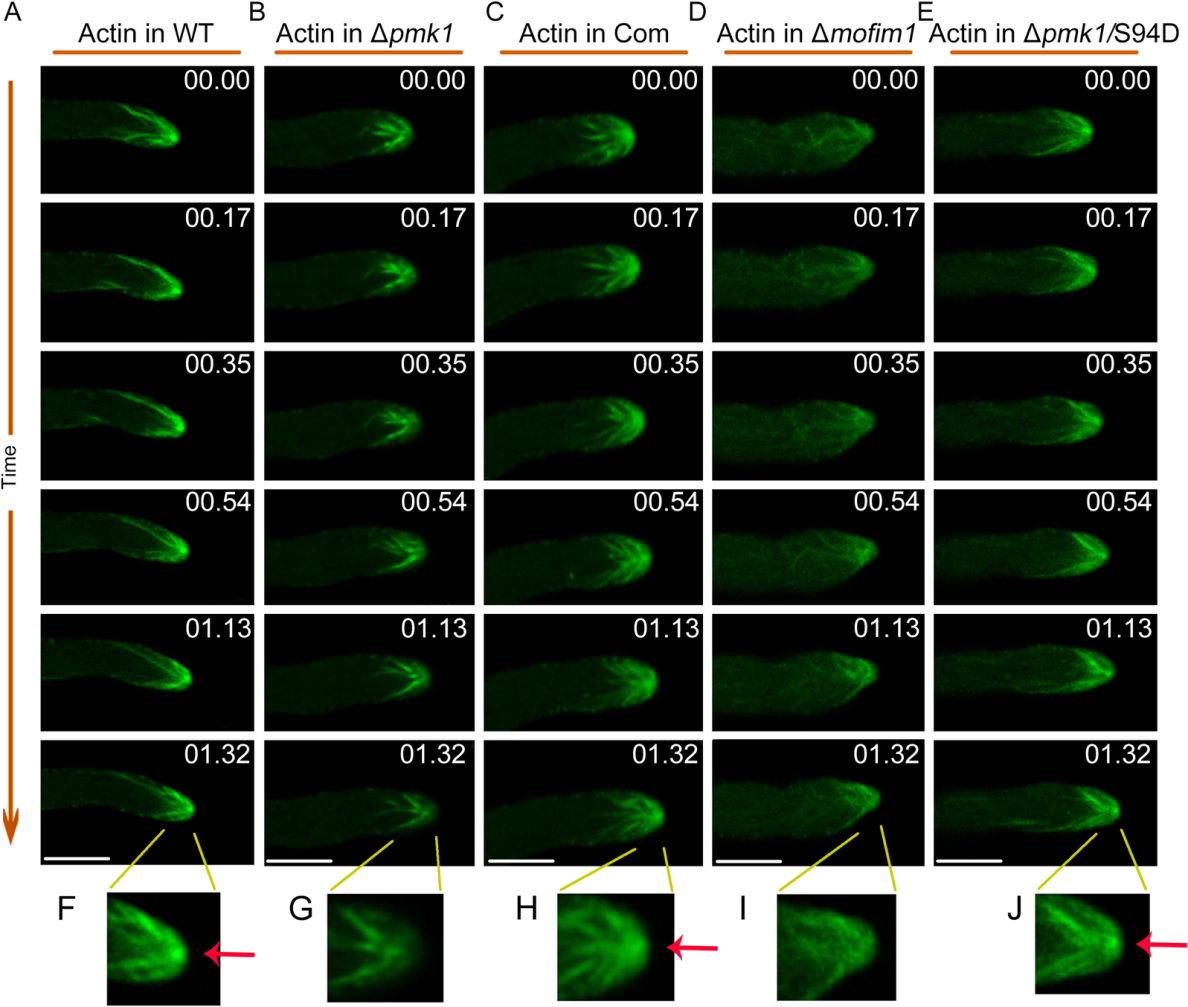
Actin organization in Δ*pmk1* and Δ*Mofim1.* The hyphae were labelled with Lifeact-GFP to visualize the actin cytoskeleton. Time-lapse images showing actin dynamics in growing hyphae of the WT (**A**), Δ*pmk1* (**B**), complemented strain Δ*pmk1* (**C**), Δ*mofim1* (**D**) and S94D (**E**). The red arrows indicate the presence of actin at Spk during polar growth. The numbers at the top right corner indicate the timestamps (min:s). Bars = 2 μm

Fimbrin is an actin-related protein that can facilitate the formation of actin bundles. We then investigated actin organization in both Δ*pmk1* and Δ*mofim1.* We expressed the actin-labelling peptide Lifeact in combination with GFP in *M. oryzae* strains. Through live-cell imaging, we found that actin was dynamically assembled at the hyphal tip in the hyphae of the WT. We observed that actin accumulated at the hyphal tip where Spk was located, and some actin cables connected to Spk (Fig. 5A and C, Supplemental Movies 3 and 5). However, in both Δ*pmk1* and Δ*mofim1*, we found that there was no stable actin accumulation in the Spk region (Fig. 5B and D, Supplemental Movies 4 and 6). In addition, when S94D was expressed in Δ*pmk1*, actin accumulation at Spk was partially restored (Fig. 5E, Supplemental Movie 7). The enlarged regions of the hyphal tips of these *M. oryzae* strains further showed that actin was distributed at Spk (Fig. 5F-J).

### S94D expression could restore the hyphal growth of Δ*pmk1*

**Fig. 6:**
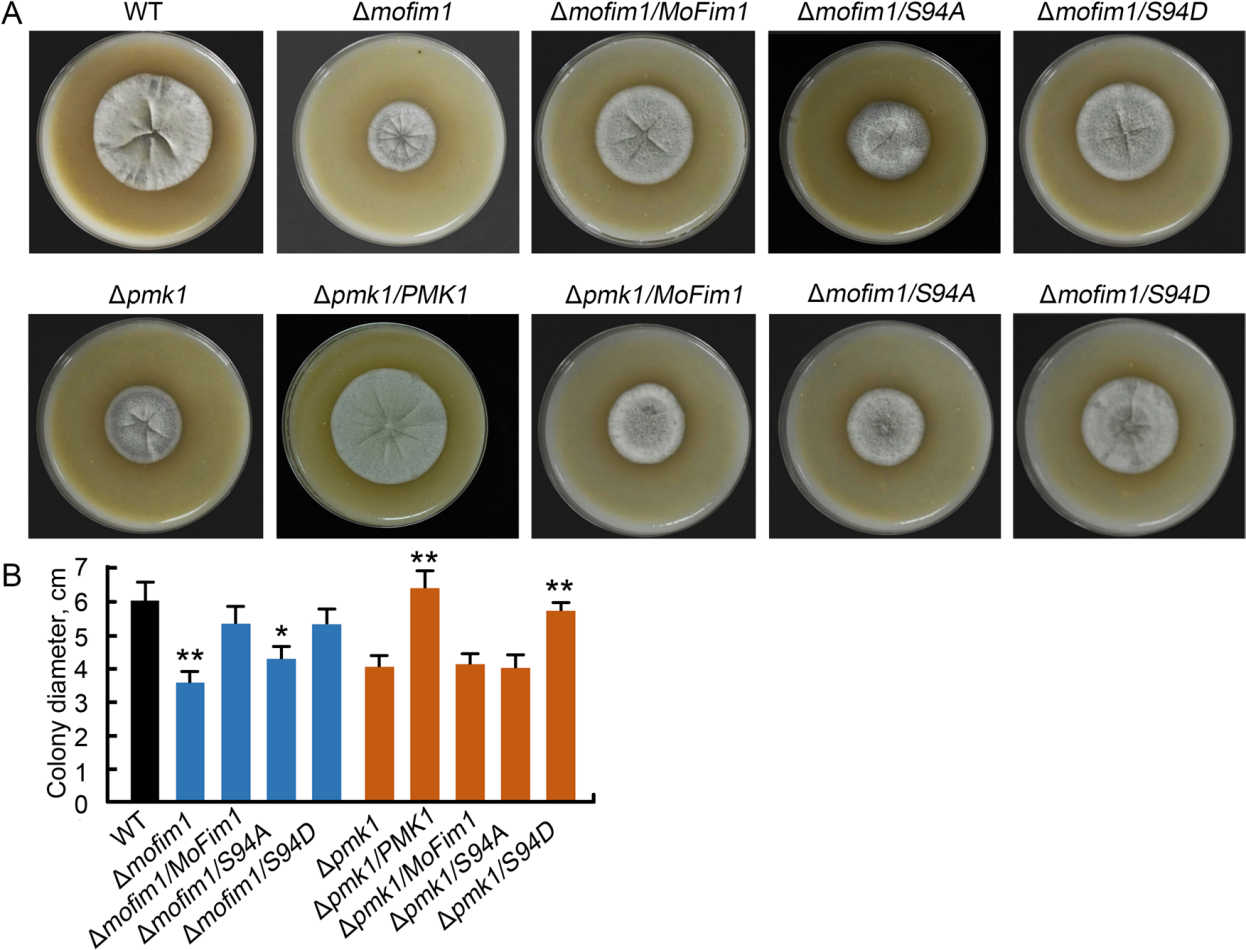
S94D has a function in rescuing the hyphal growth defect of Δ*pmk1.* **A** Five days cultured *M. oryzae* on SRB medium including the WT, Δ*mofim1*, the complemented strain of Δ*mofim1* by expressing ofum *MoFim1*, *S94A*, *S94D*, Δ*pmk1*, Δ*pmk1* expressing of *PMK1*, *MoFim1*, *S94A*, *S94D*. **B** Colony diameter of the indicated *M. oryzae* strains in (**A**). The experiments were repeated three times with similar results. The error bars represent SD (*n* = 20), and the asterisks (**) represent significant differences (*p* < 0.01)

As MoFim1 is required for the hyphal growth of *M. oryza*e, we then observed the growth of the Δ*pmk1*, Δ*mofim1* and their complementary strains. We cultured these *M. oryzae* strains on straw rice bran (SRB) medium. The results showed that the Δ*mofim1* exhibited a slower growth rate than the WT (Fig. 6). Additionally, expression of *MoFim1* and *S94D* driven by the native promoter of *MoFim1* in Δ*mofim1* rescued the phenotype (Fig. 6). The growth of the Δ*pmk1* also decreased compared with that of the WT and the complemented strain. We also expressed *MoFim1s*, including *MoFim1*, *S94A* and *S94D*, in Δ*pmk1.* We found that the expression of *MoFim1* and *S94A* could not reverse the growth-inhibiting phenotype, but S94D could increase the growth of Δ*pmk1*. The statistical analysis further supported these conclusions (Fig. 6B).

### S94D partially rescues Δ*pmk1*-induced appressorium formation failure

**Fig. 7:**
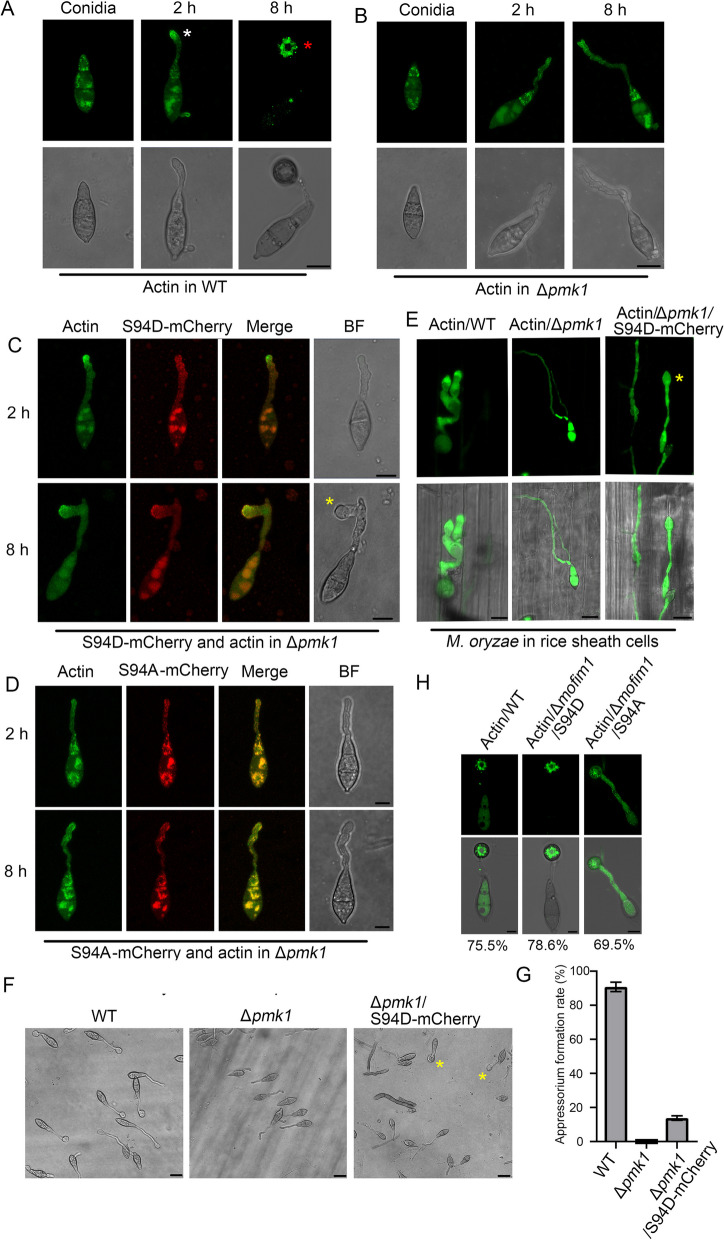
S94D could partially rescuing the failure formation of an appressorium in Δ*pmk1*. *M. oryzae* conidia of the WT (**A**), Δ*pmk1* (**B**), Δ*pmk1* expressing S94D-mCherry (**C**) and Δ*pmk1* expressing S94A-mCherry (**D**) were cultured on hydrophobic glass for 2 h to develop a germ tube and for 8 h to form an appressorium. Lifeact-GFP was expressed in these *M. oryzae* strains to visualize the actin cytoskeleton. The white arrow indicates the dense actin network at the germ tube. The red arrow indicates an actin ring present in the appressorium cell. The yellow arrow indicates a smaller appressorium in the Δ*pmk1* expressing S94D-mCherry. Additionally, Δ*pmk1* expressing S94A-mCherry could not develop an appressorium-like structure at the tip of the germ tube. Bars = 5 µm. **E** Conidia of the WT, Δ*pmk1*, and Δ*pmk1* strains expressing S94D-mCherry were used to infect the rice sheath. Images were obtained after 12 h of infection. The yellow arrow indicates a smaller appressorium in the Δ*pmk1* expressing S94D-mCherry. Bars = 10 µm. **F**, Images of the appressorium of the WT, Δpmk1 and Δpmk1 expressing S94D-mCherry. Bars = 10 µm. **G** Appressorium formation rate analysis for the WT, Δ*pmk1* and Δ*pmk1* expressing S94D-mCherry. Asterisks indicate appressorium. The error bars represent SD (*n* = 100). **H** Actin assembly in the appressorium of WT and the S94A or S94D complemented Δ*mofim1* stains. The numbers indicate the percentage of the cell presenting the actin arrangement shown in figure (*n* = 50). The experiments repeated for three times. Bars = 5 μm

We next studied whether S94D could rescue the infection defects of Δ*pmk1.* We collected actin-labelled conidia from the WT, Δ*pmk1*, and Δ*pmk1* strains expressing S94D-mCherry and S94A-mCherry. After induction on hydrophobic glass for 2 h, these conidia developed an elongated germ tube. With respect to the WT, a dense actin network accumulated at the tip of the germ tube (Fig. 7A, middle panel). However, in the Δ*pmk1*, the fluorescence in the actin network was much weaker in the tip region of the germ tube than in the WT (Fig. 7B, middle panel). The expression of S94D-mCherry but not S94A-mCherry in the Δ*pmk1* strain recovered dense actin accumulation at the germ tube tip (Fig. 7C and D). Furthermore, after 8 h of induction, the WT successfully developed an appressorium, and an actin ring appeared in the cell (Fig. 7A, right panel). Compared with the WT, the Δ*pmk1* could not form an appressorium, and no actin rings were observed (Fig. 7B, right panel). Interestingly, in the Δ*pmk1* strain that expressed S94D-mCherry but not S94A-mCherry*,* the tip of the germ tube usually expanded to form a smaller appressorium. Additionally, the fluorescence of S94D-mCherry and actin was distributed at the periphery of these cells (Fig. 7C). These results indicated that S94D contributed to the development of an appressorium for Δ*pmk1.* We next assessed whether this appressorium could penetrate and infect rice cells. These conidia were used to infect the rice sheath cells. After 12 h of infection, the WT *M. oryzae* penetrated the rice sheath cells (Fig. 7E, left panel). However, the Δ*pmk1* expressing S94D-mCherry could not penetrate into the rice cells even though it formed an appressorium-like structure (Fig. 7E, right panel). We then calculated the appressorium formation rate and found that the expression of S94D-mCherry could induce 15% of the germ tubes to develop an appressorium-like structure in Δ*pmk1* (Fig. 7F and G). And we further observed that in the WT and the S94D complemented Δ*mofim1* stain, it formed an actin ring in the appressorium cell. But in the S94A complemented Δ*mofim1* stain, it can not develop an intact actin ring (Fig. 7H). Taken together, these results indicated that the expression of S94D in Δ*pmk1* could partially rescue the morphological defects of Δ*pmk1* by inducing an appressorium but could not rescue the penetration defects associated with infection of host cells.

## Discussion

Rice blast disease is one of the greatest threats to global food security (Talbot 2003). The underlying biological processes of development and plant infection, including the regulation of hyphal growth, appressorium formation and invasive hyphal proliferation in plant cells, are critical for understanding the mechanism of *M. oryzae* pathogenesis. The actin cytoskeleton is a highly dynamic system that can organize distinct spatial structures in different cells of *M. oryzae.* They could assemble as actin patches and actin bundles in the growing hyphae (Li et al. 2020), actin ring in the formation of septum (Delgado-Alvarez et al. 2010) and hetero-oligomeric ring like structure in the infection cell, the appressorium to rupture the plant cell wall (Dagdas et al. 2012). Investigating the regulation of actin assembly is helpful for the development of new fungicides for fungal disease control (He et al. 2020). In the present study, we found that the actin-related protein MoFim1 may be regulated by Pmk1 through phosphorylation. This may represent a new regulation of fimbrin mediated actin organization pattern.

Fimbrin is a major regulator of actin organization, is conserved among eukaryotes and plays important roles in a variety of cellular processes (Skau et al. 2011). Fimbrin can be phosphorylated by a kinase. For example, budding yeast fimbrin can be phosphorylated by cyclin-dependent kinase 1 (Cdk1) at threonine 103 to stabilize the N-terminal domain of fimbrin and modulate F-actin binding to regulate actin cable assembly (Miao et al. 2016). In *M. oryzae*, fimbrin localizes to the hyphal tip region (Gupta et al. 2015) and is required for hyphal polar growth (Li et al. 2020). We further determined that *M. oryzae* fimbrin could be phosphorylated by Pmk1 at site Ser94 (Figs. 2 and 3). This is a new phosphorylation site for fimbrin. Considering that S94D could rescue the actin organization and hyphal polar growth of Δ*pmk1* (Figs. 5 and 6), we believe this site is critical for the physiological functions of fimbrin. Furthermore, we found that the mutation of Ser94 significantly decreased actin bundling activity (Fig. 3). In addition, in the Δ*pmk1*, MoFim1 did not localize to the Spk region at the hyphal tip (Fig. 4); thus, we speculate that Ser94 is critical for the Spk distribution of MoFim1 and for protein activity in actin bundling.

Knockout of *PMK1* in *M. oryzae* was first reported to prevent the formation of appressorium and invasive growth in rice plants (Xu and Hamer 1996). Pmk1 was subsequently revealed to be responsible for the control of the hyphal constriction required for fungal growth to penetrate across the plasmodesma, enabling the invasive hyphae to extend from one rice cell to the neighboring cell (Sakulkoo et al. 2018). Recently, it was further revealed that Pmk1 can phosphorylate the Hox7 homeobox transcription factor, which activates a gene expression network to regulate *M. oryzae* cell division, autophagy, and turgor generation. Pmk1 can also regulate actin cytoskeletal reorganization by phosphorylating the transcriptional regulator Mst12 (Oses-Ruiz et al. 2021). In this study, we found that Pmk1 could also directly interact with the actin-associated protein MoFim1 and regulate its phosphorylation at Ser94 (Figs. 2 and 3). Pmk1 both regulated MoFim1 activity during actin bundling and its localization (Figs. 3 and 4, Supplemental Movies 1 and 2). Like in Δ*mofim1*, actin in the Δ*pmk1* hyphal tip region was aberrantly organized, and especially at Spk, actin could not be always maintained (Fig. 5B and D, Supplemental moves 4 and 6). Considering that S94D could reverse actin organization at the hyphal tip (Fig. 5E, Supplemental move 7) and hyphal growth defects in Δ*pmk1* (Fig. 6), we believe that the regulatory effect of Pmk1 on actin organization was associated with MoFim1. Pmk1 is a critical switch in *M. oryzae* development and infection and can activate a network of genes (Oses-Ruiz et al. 2021). Thus, in our study, MoFim1 alone (and even S94D) could not completely rescue the infection failure of Δ*pmk1* (Fig. 7), although it partially rescued the morphological defects of Δ*pmk1* in inducing an appressorium. Overall, our study provides new evidence indicating how Pmk1 regulates actin cytoskeleton organization during *M. oryzae* development and pathogenesis.

## Materials and methods

### Strains and culture conditions

All *M. oryzae* isolates used in this study were derived from the sequenced WT strain Y34 (this strain was kindly provided by Prof. LiHuang Zhu, Institute of Genetics and Developmental Biology, Chinese Academy of Sciences) (Xue et al. 2012). The wild-type, Δ*pmk1*, Δ*mofim1* and complemented strains were cultured at 28 °C on complete medium (CM) plates. For conidia production, these strains were maintained in straw rice bran (SRB) medium at 28 °C for 7 days in the dark.

### Targeted gene deletion and mutant complementation

The Δ*pmk1* and Δ*mofim1* deletion mutants were generated according to previous methods (Liu et al. 2022). Briefly, two approximately 1.5 kb fragments of the sequences flanking *PMK1* were amplified with two primer pairs. The obtained DNA fragments were ligated upstream and downstream of the hygromycin gene. Then, the recombinant DNA was infused into the pGKO vector (Zhou et al. 2017) via homologous recombination cloning (ClonExpress MultiS One Step Cloning Kit, Vazyme Biotech, C112). The protoplasts of wild-type Y34 were transformed with pGKO-PMK1 for targeted gene deletion.

To construct plasmids expressing *PMK1-mCherry* and *MoFim1-GFP*, approximately 1.5 kb of the native promoter region from the *M. oryzae* genome was amplified and cloned and inserted into the pKNTG expression vector (Zheng et al. 2016). All the constructs were cloned by homologous recombination (ClonExpress MultiS One Step Cloning Kit, Vazyme Biotech, Nanjing, China; C112); all the primers with restriction enzyme sites are listed in Supplemental Table S1.

### Pathogenicity assay

*M. oryzae* conidia were harvested from 10-day-old SRB agar cultures with sterile water. The samples were filtered through Miracloth and resuspended to a concentration of 5 × 10^4^ spores/ml in a 0.2% (w/v) gelatin solution (Li et al. 2017b, 2019). Then, the conidia were injected into 20-day-old cultured rice (*Oryza sativa* *cv*. Nipponbare) sheaths. The infected rice sheaths were maintained under humid conditions at 28 °C. The leaf sheaths were observed under a Zeiss880 confocal microscope.

For infection of the rice leaves, the same region of *M. oryzae* cultured in SRB media was used to infect punched rice leaves (the third leaf of each seedling, cv. Nipponbare). The inoculated plants were kept in a growth chamber at 28 °C with 90% humidity and a 12 h/12 h light/dark cycle. Lesions from rice leaves were observed after 5 days (Zhang et al. 2011).

### Co-IP analysis

To generate the GFP tag constructs, DNA fragments, including GFP MoFim1-GFP, were cloned and inserted into the vector pSulPH (Li et al. 2020, 2017c). To generate mCherry tag constructs, mCherry and PMK1-mCherry DNA fragments were amplified and inserted into the vector pKNTG. All of the resulting GFP-tag and mCherry fusion constructs were confirmed by sequencing analysis and subsequently transformed into the Y34 strain in pairs. *M. oryzae* mycelia were cultured in CM liquid medium. The hyphae were ground into powder in liquid nitrogen and resuspended in 1 ml of extraction buffer (50 mM Tris–HCl (pH 7.5), 150 mM NaCl, 10% glycerol, 1 mM DTT, 1 mM EDTA, and 1 × protease inhibitor cocktail (Sigma, S8830)). After centrifuging for 16,000 g at 4 °C for 30 min, the supernatants were incubated with 10 μL of α-GFP-Trap-fused Magnetic Agarose (ChromoTek, GNA-25–500) for 1 h. The beads were subsequently washed four times with PBS. After the last centrifugation, the PBS was removed completely. Then, 20 μL of SDS‒PAGE sample buffer was added, and the beads were boiled for 10 min. The presence of the PMK1-mCherry or MoFim1-GFP was detected by immunoblotting for GFP (Abmart, P60046S) or mCherry (Abmart, P60056S).

### Phosphorylated-mass spectrometric analysis

The phosphorylated-mass spectrometric analysis was based on the method described previously (Yan et al. 2018). Briefly, *M. oryzae* mycelia in WT or Δ*pmk1-*expressing MoFim1-GFP were cultured in fresh CM liquid medium. The collected MoFim1-GFP *M. oryzae* mycelia were subsequently ground to powder in liquid nitrogen. The powder was fixed with three volumes of protein extraction buffer (50 mM Tris–HCl, pH 7.5; 150 mM NaCl; 10% glycerol; 1 mM DTT; 1 mM EDTA; and 1 × protease inhibitor cocktail (Sigma, S8830)) for 1 h (Gupta et al. 2015). The samples were subsequently centrifuged at 12,000 rpm at 4 °C for 30 min, after which the pellets were discarded. The supernatant was incubated with 20 μL of α-GFP-Trap-fused Magnetic Agarose (ChromoTek, GNA-25–500) for 1 h. The beads were subsequently washed four times with PBS. After the last centrifugation, the PBS was removed completely. Then, 20 μL of SDS‒PAGE sample buffer was added, and the beads were boiled for 10 min. The soluble samples were subjected to SDS‒PAGE for 10–15 min. Then, the gel was cut (approximately 1 cm) and proteins were excised and collected for digestion with trypsin at 37 °C for 18 h using a trypsin/substrate ratio of 1:50. The resulting peptides were analysed through high-resolution mass spectrometry (SCIEX, USA ZenoTOF 7600). Phosphopeptides were further enriched by IMAC beads (Phos-Select IronAffinity Gel; Sigma‒Aldrich).

### BiFC experiment

To construct the plasmids for the BiFC experiments, the *PMK1* gene with a native promoter was cloned and inserted into the C-terminus of YFP in the PKNTG vector containing a hygromycin resistance gene. The *MoFim1* gene with a native promoter was cloned and inserted into the vector PBHT2 containing the bleomycin resistance gene to generate the MoFim1-YFP plasmid. The two plasmids were subsequently transformed into Y34 protoplasts. The obtained *M. oryzae* clones were examined using fluorescence microscopy (Zeiss880 microscope). All the constructs were generated via homologous recombination cloning (ClonExpress MultiS One Step Cloning Kit; Vazyme Biotech). The primers used are listed in Supplemental Table 1.

### Yeast two-hybrid assay

Full-length cDNAs of *MoFim1*, *ABD1*, *EF* and *ABD2* were cloned and inserted into the pGADT7 (AD) vector. Full-length cDNAs of the *PMK1* gene were inserted into the pGBKT7 (BD) vector. To examine the interactions between Pmk1 and MoFim1, the AD and BD constructs were coexpressed in the yeast strain AH109, and clones were screened on SD-Trp-Leu media. Then, the transformants were isolated and grown on SD-Trp-Leu-His-Ade media. The sequences of primers used are listed in Supplemental Table 1.

### In vitro protein purification and F‐actin binding/bundling assay

The cDNA fragments containing the *MoFim1*, *S94D*, *S94A*, *S117A* or *PMK1* ORF were infused into the bacterial expression vector pET-28a (Novagen/Merck) with the enzyme sites BamH1 and SalI to produce His-tagged MoFim1s-GFP fusion proteins, including MoFim1, S94A, S94D, S117A and S117D. The constructs were subsequently transformed into Transetta (DE3) chemically competent cells (TransGen, CD801-02). The expression of the fusion proteins was induced by 0.5 mM IPTG for 5 h at 28 °C. These recombinant proteins were purified using ProteinIso Ni–NTA resin (TransGen, DP101-01) according to the manufacturer’s instructions. All primers and restriction enzyme sites are listed in Table S1.

In vitro phosphorylation analysis was performed according to previous methods (Feng et al. 2022). The purified Pmk1 alone or in combination with the MoFim1, S94A, or S117A proteins was mixed in kinase reaction buffer (100 mM PBS, 1 mM ascorbic acid (pH 7.5), and 10 mM MgCl_2_ with 50 µM ATP at 25°C for 1 h. Then, tenfold cold acetone was added to terminate the reaction. These reaction samples were diluted with 1 × SDS-loading buffer (TransGen, DL101-02). The proteins were separated by SDS‒PAGE. Then, the phosphorylation of MoFim1 was detected using a commercial antibody that recognizes phosphorylated Ser and Thr (ECM Biosciences, PP2551, 1:1,000 dilution).

Visualization of actin filaments in the presence of the recombinant proteins was performed by fluorescence microscopy as reported previously (Han et al. 2013). Briefly, prepolymerized rabbit F-actin (1 μM) (Cytoskeleton, BK037) was incubated with MoFim1 and its corresponding site mutant proteins (1 μM) at room temperature for 30 min and labelled with Alexa561-phalloidin (Aladdin, P287444). Images were obtained under a confocal microscope (LSM880; Zeiss) at 561 nm.

### Observation of fluorescent signals by live-cell imaging and actin analysis

Live-cell imaging experiments were conducted as previously described (Abubakar et al. 2023). Briefly, the mycelia were cultured in liquid CM for 24 h. The mycelia were placed upside down on a glass slide, with the fungal hyphae directly touching the surface of the slide. Then, the growing hyphae were observed under a confocal microscope (LSM880; Zeiss) equipped with an Airyscan detector for time-lapse imaging. The laser lines included GFP (using argon laser excitation at 488 nm and emission spectra at 500–540 nm) and mCherry (using neon laser excitation at 561 nm and emission spectra at 600–640 nm).

### Supplementary Information

**Additional file 1: Supplemental Figure 1. ***M. oryzae* strains used in the co-IP assay. *GFP*, *mCherry* driven by toxA promoter and *PMK1-mCherry*, *MoFim1-GFP* driven by their native promoters were expressed as indicated, and were used in the co-IP experiment. Bars = 5 µm. **Supplemental Figure 2. **Subcellular distribution of Pmk1. *PMK1-mCherry *driven by its native promoter was expressed in actin-labelled *M. oryzae. *Pmk1-mCherry protein signals were observed in the conidia, germ tube, appressorium and hyphae via confocal microscopy. Bars = 10 µm. **Supplemental Figure 3. **Targeted *PMK1* deletion in *M. oryzae. *Schematic illustration of the deletion of *PMK1 *in *M. oryzae *(A). PCR analysis of the *PMK1 *deletion mutants with the indicated primer pairs (B). Lanes 1, 2, and 3 indicate the mutant strains, and lane 4 indicates the WT strain. **Supplemental Table 1.** The nucleotides highlighted in red indicate the enzyme site for construction.

**Additional file 2: Supplemental Movie 1. **Distribution of MoFim1-GFP in the WT growing hypha. MoFim1-GFP driven by the native MoFim1 promoter was expressed in WT *M. oryzae*. Time-lapse images were obtained with a high-resolution live-cell imaging system. The numbers at the top right corner indicate the timestamps (min:s). Bars = 2 µm.

**Additional file 3: Supplemental Movie 2. **Distribution of MoFim1-GFP in the Δ*pmk1* growing hypha. MoFim1-GFP driven by the MoFim1 native promoter was expressed in the Δ*pmk1.* The time-lapse images were obtained under a high-resolution live-cell imaging system. The numbers at the top right corner indicate the timestamps (min:s). Bars = 2 µm.

**Additional file 4: Supplemental Movie 3-7. **Dynamic actin assembly in growing hyphae. Lifeact-GFP was expressed in the WT (Supplemental Movie 3), Δ*pmk1 *(Supplemental Movie 4), Δ*pmk1 *complemented (Supplemental Movie 5), Δ*mofim1 *(Supplemental Movie 6) and Δ*pmk1 *expressing S94D (Supplemental Movie 7). These representative videos are based on data from 20 mycelia in three independent experiments for each. The numbers at the top right corner indicate the timestamps (min:s). Bars = 5 µm.

## Data Availability

All data generated or analyzed during this study are included in this published article.
