# Supplementary material for: Fimbrin associated with Pmk1 to regulate the actin assembly during Magnaporthe oryzae hyphal growth and infection
Source: Stress Biol. 2024 Jan 22;4(1):5. doi: 10.1007/s44154-023-00147-5 (PMC10803693; doi:10.1007/s44154-023-00147-5)
Supplement: Supplementary file 1 — Additional file 1: Supplemental Figure 1. M. oryzae strains used in the co-IP assay. GFP, mCherry driven by toxA promoter and PMK1-mCherry, MoFim1-GFP driven by their native promoters were expressed as indicated, and were used in the co-IP experiment. Bars = 5 µm. Supplemental Figure 2. Subcellular distribution of Pmk1. PMK1-mCherry driven by its native promoter was expressed in actin-labelled M. oryzae. Pmk1-mCherry protein signals were observed in the conidia, germ tube, appressorium and hyphae via confocal microscopy. Bars = 10 µm. Supplemental Figure 3. Targeted PMK1 deletion in M. oryzae. Schematic illustration of the deletion of PMK1 in M. oryzae (A). PCR analysis of the PMK1 deletion mutants with the indicated primer pairs (B). Lanes 1, 2, and 3 indicate the mutant strains, and lane 4 indicates the WT strain. Supplemental Table 1. The nucleotides highlighted in red indicate the enzyme site for construction. [file 44154_2023_147_MOESM1_ESM.pdf]

Fimbrin associated with Pmk1 to regulate the actin assembly during *Magnaporthe oryzae* hyhpal growth and infection

Yuan-Bao Li[1,2#], Ningning Shen[1,2#], Xianya Deng[1,4], Zixuan Liu[1,4], Shuai Zhu[1,3], Chengyu Liu[1,3], Dingzhong Tang[1,2*], and Li-Bo Han[1,2*]

*** Correspondence:**

Li-Bo Han (Email: hanlibo@fafu.edu.cn).

Dingzhong Tang (Email: dztang@fafu.edu.cn);

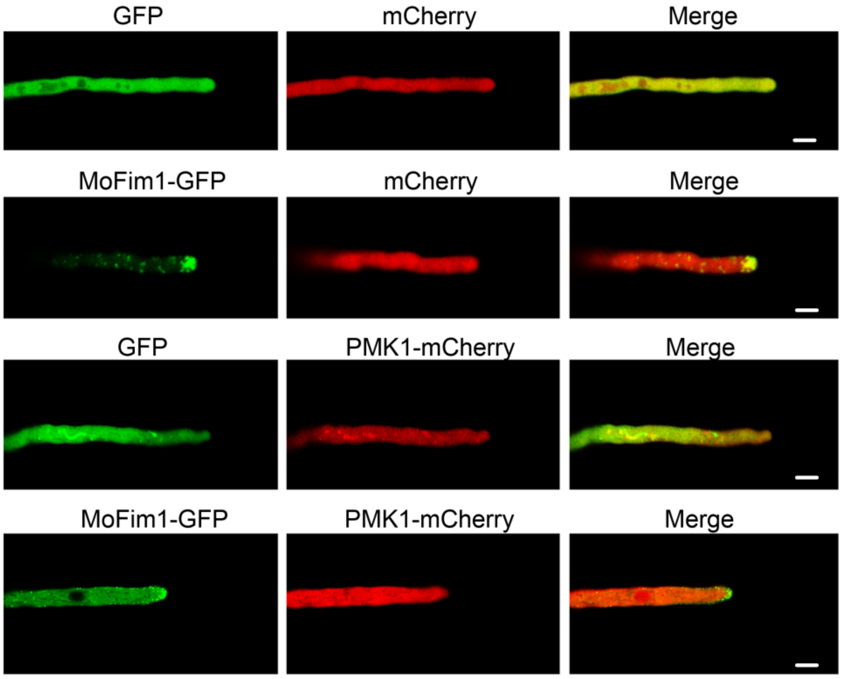

Supplemental Figure 1. *M. oryzae* strains used in the co-IP assay

GFP, *mCherry* driven by toxA promoter and *PMK1-mCherry*, *MoFim1-GFP* driven by their native promoters were expressed as indicated, and were used in the co-IP experiment. Bars = 5 µm.

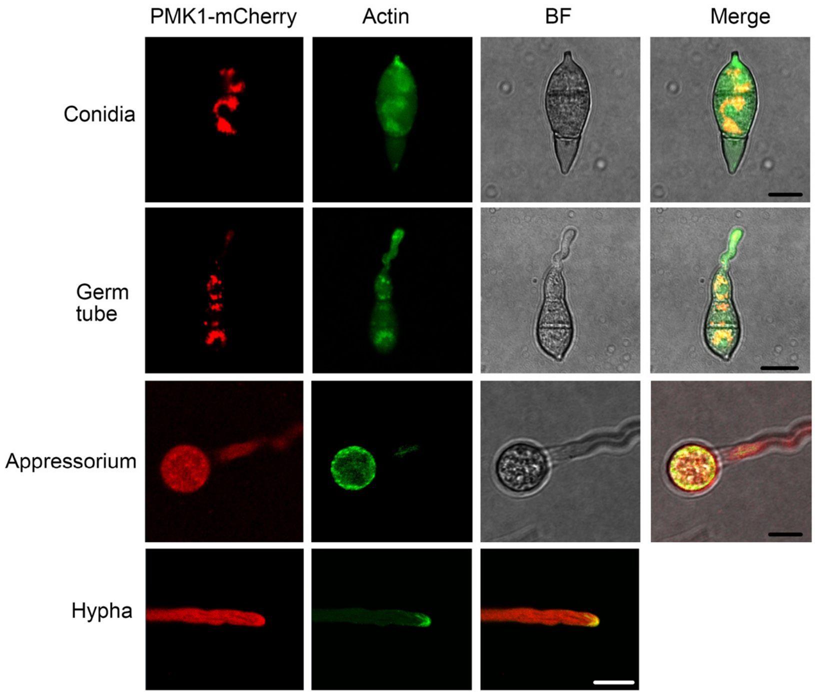

Supplemental Figure 2. Subcellular distribution of Pmk1

PMK1-mCherry driven by its native promoter was expressed in actin-labelled *M. oryzae*. Pmk1-mCherry protein signals were observed in the conidia, germ tube, appressorium and hyphae via confocal microscopy. Bars = 10 µm.

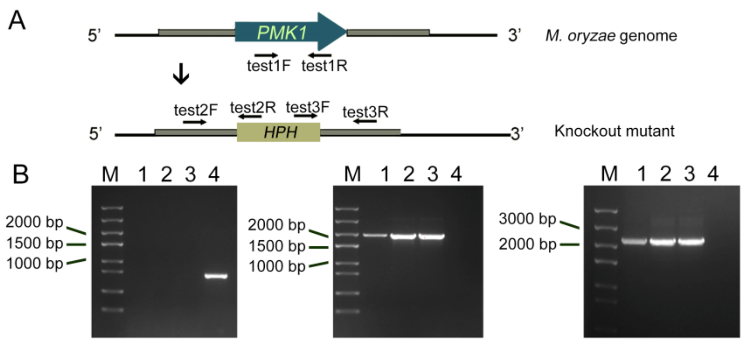

Supplemental Figure 3. Targeted *PMK1* deletion in *M. oryzae*

Schematic illustration of the deletion of *PMK1* in *M. oryzae* (A). PCR analysis of the *PMK1* deletion mutants with the indicated primer pairs (B). Lanes 1, 2, and 3 indicate the mutant strains, and lane 4 indicates the WT strain.

Supplemental Table1

The nucleotides highlighted in red indicate the enzyme site for construction.

For knock out*PMK1*	
PMK1-KO-1F	GGTGGCGGCCGCTCTAGACAACTTCACGTAGCGATGTGATCA
PMK1-KO-1R	CAAAAATGCTCCTTCAATCTAGACTTGGTGGATTGGCGCGAGAC
PMK1-KO-3F	GGGTTCGCAAAGATAAAAGCTTTAATGCGGCGAGCGGTCCATGGTC
PMK1-KO-3R	GGTCGACGGTATCGATAAGCTTCTTACGACATTCACGCCGATGACG
PMK1-test-1F	GCGACTTAATGGCTGACATG
PMK1-test-1R	GACTTGATACCATAGTAGTC
PMK1-test-2F	GTATTGTATTGCACGGCCG
PMK1-test-2R	GCTGATCTGACCAGTTGC
PMK1-test-3F	GTCGATGCGACGCAATCGT
PMK1-test-3R	CCTTGAGACACAAGGCACGAGC
For localization	
PBN-GFP-F	ACCGTCAAACCGAGCTCGGTACCATGGTGAGCAAGGGCGAGGAG
PBN-GFP-R	GTTTGAACGATCGGATCCCTAGTCGACCTTGTACAGCTCGTCCAT
PBH-mCherry-F	CCGAGCTCGGTACCGGATCCATGGTGAGCAAGGGCGAGG
PBH-mCherry-R	CAAATGTTTGAACGATCGGATCCCTACTTGTACAGCTCGTCCATGCC
PBH-NaproPMK1-mCherry-F	GCTATGACCATGATTACGAATTCCAACTTCACGTAGCGATGTGATCA
PBH-NaproPMK1-mCherry-R	CCTTGCTCACCATGGATCCGGTACCCCGCATAATTTCCTGGTAG

PBN-NaproMoFim1-GFP-F	GCTATGACCATGATTACGAATTCGGCGGAGCCATCAAGTCTGT
PBN-NaproMoFim1-GFP-R	CCTCGCCCTTGCTCACCATGGTACCAGCCATCTTTTCATGCGTCGC
YC-PMK1-F	GCCACAACATCGAGGACGTCGACATGTCTCGCGCCAATCCACC
YC-PMK1-R	GAACGATCGGATCCCTAGTCGACCCGCATAATTTCCTGGTAG
YN-MoFim1-F	CCGTCAAACCGAGCTCGGTACCATGAACGTTCTCAAACTTCAGAG
YN-MoFim1-R	GCACGCTGCCGCCCATGGTACCCGCCATCAGGGAACCAATAAACG
For biochemical experments	
pET-28a- GFP-F	CAGCAAATGGGTCGCGGATCCATGGTGAGCAAGGGCGAGGAGCTGT
pET-28a- GFP-R	GTGCTCGAGTGCGGCCGCAAGCTTCTTGTACAGCTCGTCCATGCCGT
pET-28a-GFP-MoFim1-F	GGACGAGCTGTACAAGAAGCTTATGAACGTTCTCAAACTTCAGAG
pET-28a-GFP-MoFim1-R	TCGAGTGCGGCCGCAAGCTTTCAAGCCATCTTTTCATGCGTC
pET-28a -S94A-1	GGACGAGCTGTACAAGAAGCTTATGAACGTTCTCAAACTTCAGAG
pET-28a-S94A-2	GCCGCCGCCCGCAAGACGCT TTTGCGCGGG GGAGGACTGG CGC
pET-28a-S94A-3	GCGCCAGTCCTCCCCCGCGCAAAAGCGTCTTGCGGGCGGCGGC
pET-28a-S94A-4	GTGGTGGTGGTGGTGCTCGAGTCAAGCCATC TTTTCATGCG TC
pET-28a-S94D-1	GGACGAGCTGTACAAGAAGCTTATGAACGTTCTCAAACTTCAGAG
pET-28a-S94D-2	GCCGCCGCCGTCAAGACGCT TTTGCGCGGG GGAGGACTGG CGC
pET-28a-S94D-3	GCGCCAGTCCTCCCCCGCGCAAAAGCGTCTTGACGGCGGCGGC
pET-28a-S94D-4	GTGGTGGTGGTGGTGCTCGAGTCAAGCCATCTTTTCATGCG TC
For Y2H	

AD-MoFim1-F	GTACCAGATTACGCTCATATGATGAACGTTCTCAAACTTCAGAG
AD-MoFim1-R	CAGCTCGAGCTCGATGGATCCTCAAGCCATCTTTTCATGCGTC
BD-PMK1-F	GCCATGGAGGCCGAATTCATGTCTCGCGCCAATCCACCAAG
BD-PMK1-R	CGCTGCAGGTCGACGGATCCTTACCGCATAATTTCCTGGTAG
